# *Bacillus thuringiensis* Is an Environmental Pathogen and Host-Specificity Has Developed as an Adaptation to Human-Generated Ecological Niches

**DOI:** 10.3390/insects5010062

**Published:** 2013-12-24

**Authors:** Ronaldo Costa Argôlo-Filho, Leandro Lopes Loguercio

**Affiliations:** Department of Biological Sciences, State University of Santa Cruz (UESC), Rod, Ilhéus-Itabuna, Km-16, Ilhéus-BA 45662-900, Brazil; E-Mail: leandro@uesc.br

**Keywords:** *Bacillus* ecology, multiple hosts, virulence factors, pathogenicity

## Abstract

*Bacillus thuringiensis* (*Bt*) has been used successfully as a biopesticide for more than 60 years. More recently, genes encoding their toxins have been used to transform plants and other organisms. Despite the large amount of research on this bacterium, its true ecology is still a matter of debate, with two major viewpoints dominating: while some understand *Bt* as an insect pathogen, others see it as a saprophytic bacteria from soil. In this context, *Bt*’s pathogenicity to other taxa and the possibility that insects may not be the primary targets of *Bt* are also ideas that further complicate this scenario. The existence of conflicting research results, the difficulty in developing broader ecological and genetics studies, and the great genetic plasticity of this species has cluttered a definitive concept. In this review, we gathered information on the aspects of *Bt* ecology that are often ignored, in the attempt to clarify the lifestyle, mechanisms of transmission and target host range of this bacterial species. As a result, we propose an integrated view to account for *Bt* ecology. Although *Bt* is indeed a pathogenic bacterium that possesses a broad arsenal for virulence and defense mechanisms, as well as a wide range of target hosts, this seems to be an adaptation to specific ecological changes acting on a versatile and cosmopolitan environmental bacterium. *Bt* pathogenicity and host-specificity was favored evolutionarily by increased populations of certain insect species (or other host animals), whose availability for colonization were mostly caused by anthropogenic activities. These have generated the conditions for ecological imbalances that favored dominance of specific populations of insects, arachnids, nematodes, *etc.*, in certain areas, with narrower genetic backgrounds. These conditions provided the selective pressure for development of new hosts for pathogenic interactions, and so, host specificity of certain strains.

## 1. Introduction

*Bacillus thuringiensis* (*Bt*) is a widespread endospore-forming bacteria with a complex life cycle, which has been commonly found in soil, water, plants, stored cereals and dead insects. It is classified as a Gram-positive, facultative anaerobic and its main feature is the production of crystalline proteinaceous inclusions during the stationary and sporulation growth phases [1,2,3]. According to Lecadet *et al.* [4] and Xu and Côté [5], the *Bt* classification based upon biochemical properties and composition of the flagellar antigen ‘H’ contains 69 serotypes, 82 antigenic serovars and 13 antigenic subgroups, thereby demonstrating the high degree of genetic variability presented by this species. This classification, however, does not take into account the toxicity profile presented by the diverse array of *Bt* lineages isolated wordlwide, which is defined by the type(s) of toxin(s) produced. 

Due to economic necessity and ease of manipulation, the majority of *Bt*-related research has focused on direct effects against insect-pests relevant to agriculture. However, studies focusing purely on the ecology of *Bt*, including its interactions with a diverse range of organisms that occupy the same niches are scarce. The discussion about the real ecology of *Bt* has heated up in the last few years, with various researchers now referring to *Bt* as an obligate pathogen [1,2,6,7]. To shed more light on this matter, some studies have demonstrated that, as long as the *Bt* metabolic requirements are met, this bacterium grows vegetatively in a variety of environments, such as soil, leaf surfaces and other internal plant tissues, although this growth occurs in much lower levels when compared with that inside insect cadavers [8,9]. It is important to realize that we cannot consider the soil only as a general deposit for *Bt*, since it is from such environment that *Bt* can colonize rhizospheres, germinating plants and invertebrates, so that it can multiply in a sufficient level and reach appropriate places that allow proper infection of *bonafide* hosts [6,8,10]. Taking this into consideration, the concept of ‘environmental pathogen’ has emerged, referring to microbes that spend a significant portion of their lifecycle outside hosts, but can cause disease when in coming into contact with them [11]. The ability to survive and grow outside a host, a large dispersal capacity in an array of different environments, the recognized genetic plasticity that provides an adaptive arsenal to infect multiple hosts, all help explain why it is not trivial to monitor, assess and understand the ecology of *Bt*. In this review we addressed some concepts that are seldom used in *Bt* context, placing them into perspective with other common views in the attempt to stimulate critical discussion and aid in the understanding of *Bt*’s role in nature.

## 2. Niches Occupied by *Bt*

The ecology and lifestyle of *Bt* have been a target of many yet unanswered questions. Some defends that *Bt* is a soil-dwelling microorganism that obtains nutrition for its survival and reproduction in nature from decaying organic matter or roots exudates, reaching the aerial parts of plants when these germinate and emerge from soil. An opposed view states that *Bt* is a specialized pathogen that, by colonizing and killing its hosts, and multiplying in their cadavers, is then deposited in soil and plants, which thereby become natural reservoirs. In fact, according to *Bt*’s capability to survive and colonize various niches (see more below), it is reasonable to classify it as a ‘copiotrophic’ microorganism.

### 2.1. Soil Environment

Many authors classify *Bt* as a soil microorganism due to the fact that a great deal of its isolation throughout the years has been obtained from this environment (e.g., [12,13,14,15]). Others, however, consider the soil only as a storage *milieu* for *Bt* spores, as they barely germinate in these places, requiring specific nutrients and pH conditions for that [16,17,18,19]. The indigenous microbiota, as well as soil properties, such as pH, humidity, mineral and organic matter concentrations have a direct effect on *Bt*’s survival, acting positively or negatively on germination, growth, sporulation, and production of proteins [18,20,21,22,23]. These conditions, on another hand, tend to have a much lesser effect on *Bacillus cereus* (a *taxon* very closely related to *Bt*), which has shown the ability to multiply in non-sterilized soils, increasing its population up to 20% [17]. Nevertheless, under specific conditions, *Bt* spores can germinate and grow well, such as in humid, nutrient-rich soils, with pH near neutrality, even in the presence of other microbial populations [18]. Moreover, *Bt* toxins can find protection against precocious degradation in the soil, as they associate with humic acids and/or clay particles, which can help keep their toxicity for soil microbes [24,25].

*Bt* has been found as a natural soil inhabitant from several regions in the world. Areas with no history of *Bt*-based products application have shown a great diversity of H-serotype isolates with variable levels of toxicity, and persistence of *Bt* for many years after its application in areas of environmental conservation has been reported as well [12,26,27,28,29]. However, they noticed a reduction in the number of spores through time, which agrees with the idea that *Bt* hardly multiplies in this environment. Petras and Casida [30] have also observed a reduction in the initial population of spores, although it has tended to stabilization. On the other hand, the presence of insects does not guarantee the presence of *Bt* in the soil. *Bt* has been found in soils with little or no insect activity, whereas it was not found in soils with high insect activity [12]. Moreover, *Bt* apparently does not have to be an obligate microbe in its association with insects, as it grows in regular media *in vitro* [12]. Nevertheless, the chemical properties of the assessed soils, which are widely known to interphere in *Bt*’s survival [18,20,21,22,23], have not been analyzed. 

It is yet unclear which is the main form of *Bt* dispersion in the soil. It is known that its soil dispersal is limited, as it appears that very little of *Bt* populations in soil is altered by the action of rain, invertebrates or by plant growth [8,29,31,32]. However, it has been found that *Bt* spores can pass through the digestive tract of vertebrates and invertebrate organisms, reaching their feces. In these circumstances, they find favorable conditions for germination, and so, become capable of being dispersed in the environment through migration of these animals [8,33,34,35,36].

The researchers who argue that *Bt* is a soil microorganism generally believe in its saprophytic lifestyle. However, research focusing on the colonization of insects cadavers has shown that *Bt* is in fact necrotrophic, which appears to be an important feature to ensure bacterial proliferation and dispersion in the environment. Naryanan [37] demonstrated that an infected dead insect on a leaf surface caused the death of one third of the larvae around. Dubois *et al.* [38] showed that the necrotrophic stage is part of a complex life cycle of *Bt* that involves the activation and regulation of several genes that alter its metabolism after the host death. Such changes are required for survival and colonization of the host cadaver, involving the production of enzymes (proteases, lipases, esterases and chitinases) that allow not only the use of host contents, but also the breakage of cuticle for release of toxins and spores. However, the main survival factor for *Bt* seems to be the ‘kurstakin’, a lipopeptide with biosurfactant and antifungal activity that grants mobility, ability to form biofilms and improved microbial competition. Outside the concept of necrotrophism, few studies have demonstrated a saprotrophic lifestyle for *Bt*, such as growth on feces and wastewater sludge [39,40,41].

### 2.2. Epiphytic Environment—Phylloplane

*Bt* has been isolated from the phylloplane in natural or artificial ways [34,42,43,44]. It has been shown that *Bt* can reach this niche by rain splash from the soil to lower leaves [31], from soil as a result of being carried by germinating seeds [43], from animal feces such as those from insects or birds [34,36], and from dead insects [34]. There is a variation in *Bt*’s survival rates in the phylloplane that seems to be linked to the plant species. As an example of this effect, the strain HD1 was able to grow vegetatively and persist in *Trifolium hybridum* leaves [34], whereas in cotton leaves, its population disappeared entirely in few days [45]. In addition, there are some evidences indicating *Bt* as a poor leaves colonizer, being found mostly as spores in these habitats. Even with nutritionally rich leaf surface that leads to spore germination, vegetative *Bt* cells sporulates again after a few rounds of division, which confers a survival ability for long periods, even under stressing conditions, such as desiccation [42]. Despite serving as nutrient sources, leaf exudates can also affect *Bt* survival negatively; for instance, organic acids that decrease surface pH can increase mortality rates of *Bt* in this environment [18,46]. Because *Bt* demonstrates a better survival and persistence in soil than in leaves, these have been suggested to work as a secondary reservoire that aid in the recycling process of the bacterium by returning cells and spores to the soil through rain, falling leaves, feces from phytophagous and dead insects bodies [8,31,42]. Alternatively, epiphytic *Bt* may well be a transient condition between endophytic environment and insect host (see below).

### 2.3. Epiphytic Environment—Rhizosphere

Few studies have demonstrated rhizosphere colonization by *Bt*. Rabinovitch *et al.* [47] isolated a *Bt* strain from the rhizosphere of a *Ficus doliaria* tree highly toxic against the Blackfly larvae (Simuliidae), a vector of human diseases, which was not present in the area. Interestingly, the region where the tree was found had not received any prior application of a *Bt*-based product. The closely related *B. cereus* was also shown to germinate and grow in the rhizosphere of plants [48]*.* Hendricksen and Hansen [8] found a *Bt* population that was 260 times higher in the rhizosphere than in the phylloplane of the same plants. It is assumed that such a more effective colonization of *Bt* in the rhizosphere and roots surfaces might be likely due to a richer nutrient availability in these environments [49], as carbohydrates and amino acids are more abundantly released in the form root exudates [50], thereby favoring microbial growth. Nevertheless, a simply better adapted genetic set up for this particular population cannot be ruled out as a reason for this successful rhizosphere colonization.

### 2.4. Endophytic Environment

Generally, endophytic microorganisms have the ability to colonize internal plant tissues without damaging the physiological processes and the morphology of the plant, in a mutually beneficial symbiotic relationship [51], or in a neutral non-damaging form. It has been reported that bacterial endophytes, after entering and colonizing plant tissues, can reach the seeds systemically and be vertically transmitted [52,53]. Another form of transmission is the horizontal, in which environmental microbes can enter the plant body mainly through the roots [54], the epidermis (passive absorption during transpiration—Quadt-Hallmann *et al.* [53]) and stomata [55]. 

In an endophytic relationship, microbes (including *Bt*) take up necessary nutrients from the plant to survive, but compensate such activity by promoting protection of the plant against parasite attacks (by stimulation of, and direct production of toxins), and emergence of diseases (by production of antimicrobial agents and enzymes, or induction of plant immunity system [56,57]. With all these functionalities under play, the plant becomes well assisted in its growth and development [51,58,59]. Some studies in cotton, soybean, corn, sugar cane, cabbage, ricebean, gahat and lentil [50,60,61,62] have reported that *Bt* was successful in endophytic colonization, even with concomitant production of Cry toxins; the efficient *Bt* colonization of cabbage seedlings roots suggests this might be in fact the main route of its penetration in the plant. After this event, vegetative cells, spores and crystals were found in several parts of the seedlings, which characterized a complete *Bt* colonization [60]. Similarly, *Bt* was able to colonize the roots of certain legumes, which resulted in an increase of nodulation and growth of the plants [49,63]. Recent work of our group added passionfruit and cacao to this list of plants with rhizospheric/endophytic *Bt* [64,65]. Even in its vegetative stage, *Bt* produces toxins that can reduce pests or diseases attacks [66]. *Bt* can reach the interior of the plant through the roots, stomata and wounds, or through the action of hydrolytic enzymes [53,67]. It is known that Cry toxins are inactivated by UV radiation and can be washed out from the leaves by rain or irrigation [6,68,69,70]; when *Bt* grows endophytically, these adverse conditions do not occur. Recent studies, however, have evidenced that biocontrol strategies employing direct endophytic capabilities of *Bt* have been much less explored than transformation of other endophytic bacteria to express *Bt* toxins, which removes the requirements for sporulation [71,72,73,74,75].

### 2.5. Aquatic Environment

Although few studies have focused on the survival in water, they have shown such a capability for *Bt*. Ichimatsu *et al.* [39] isolated a great variety of *Bt* serovars from 50% of running (river, stream, and ditch) and still water (pond) samples in Japan. They found that 26.7% of isolates exhibited larvicidal activities against Diptera *Culex pipiens molestus* and *Clogmia albipunctata*. Such an action in aquatic organisms can be viewed as a way of promoting recycling and dispersal of *Bt* in a wider variety of environments. By assessing the survival rates of *Btk* in water samples, Menon and Mestral [76] have shown that its viability remains for large periods: around 40 days in sea water, and over 70 days in lake water, likely due to a greater nutrients availability in this case. Konecka *et al*. [27] isolated a *Bt* strain from a forest creek sample that appeared to be 24× more toxic than the *Btk* HD1 against *Cydia pomonella* (Lepidoptera) larvae. This isolate carried the *cry1B* and *cry15* genes that encode toxins against coleopteran, dipteran, and lepidopteran insects; the Cry15 is a binary toxin that has been rarely found. Further work has also demonstrated the presence of *Bt* in water samples subjected to hypochloride treatment [77,78]. Bacterial viability in aquatic environments is influenced by biological, chemical and physical factors (e.g., microbial competitors, bacteriophages, pH, aeration and nutrients); for instance, it was shown that *Bt* is capable of growth in aquatic environments that are rich in nutrients and oxigen, such as those from sewage treatment stations [39,79]. Another interesting possibility found was the use of vacuoles excreted by aquatic protozoans, such as *Tetrahymena pyriformis* [80], as nutritional source for *Bt* growth in natural environments. Taken together, these data suggest that *Bt* is also ubiquitous in aquatic environments. Since most part of water-isolated serovars were also isolated from soil and phylloplane, it is likely that they reach the water bodies through rain, percolation, floodings, wind, animal excrements, *etc.*

### 2.6. Paratenic Behavior

As it could be seen thus far, *Bt* is a versatile bacterium that occupies various niches, becoming readily accessible to a great variety of organisms. Digestion is the main access route of *Bt* to invertebrate or vertebrate animals [36]. When a microorganism colonizes a certain host in a way that it does not cause damage (disease), and this host is not indispensable to the microbe’s life, this organism is known as ‘paratenic host’. An array of vertebrates and invertebrates has shown to be colonized by *Bt*, although only few studies have gone deeper in the understanding of the ecological relationships among *Bt* and these organisms, both in the pathogenic and non-pathogenic way. For instance, Wilcks *et al.* [33] demonstrated *Bt*’s capability of colonizing the whole intestinal tract of germ-free rats, in high concentrations and in a stable form; *Bt* grew vegetatively for various generations (>90% of *Bt* population) before sporulation and elimination through their feces. Ammons *et al.* [35] reported the presence of *Bt* in rectal samples from milk cows, with indication that multiplication of *Bt* cells had occurred in the digestive tract of these animals. Zhang *et al.* [36] showed the presence of *Bt* in the intestinal tract of chickens, with the duodenum being the main portion colonized; moreover, it was verified that these animals kept releasing the bacterium through their feces for a certain time, even after removal of *Bt* from the diet. Similarly to vertebrates, *Bt* has been also isolated from fecal pellets of non-susceptible caterpillars from forests of conservation areas [44]. In another report, *Bt* germination in the alimentary tract was demonstrated in different invertebrates (e.g., earthworms), with sporulation occurring after defecation [8]. Even assuming that intestinal colonization does not occur, the fact that the *Bt* spores are able to cross the digestive tract of these animals and reach their feces does provide a nutrient-rich environment for their multiplication [34,39,41]. This ecological feature of survival, proliferation and sporulation in paratenic hosts warrants to *Bt* a wide dispersion in the environment through animals migration and defecation; such a dispersion mode is not only critical in the process of *Bt* populations recycling and multiplication in nature, but it also may help explain, at least in part, the presence of *Bt* in regions where specific host insects were not apparently found [12]*.*

Kweon *et al.* [81] isolated a *Bt* strain from fresh milk samples obtained from healthy cows udders, which were likely colonized by *Bt* from the external environment. Interestingly, this isolate was capable of working as an oral probiotic, preventing fatal pathogenic infections by bacteria in mice; the authors suggest that antibiotic production, tissue colonization, and host immune system stimulation by *Bt* turn it a strong candidate for development of an efficient probiotic product. These results are suggestive of a possible symbiotic behavior between *Bt* and other organisms [82]. 

### 2.7. Pathogenic Behavior

The microorganisms can be classified according to their specific abilities to parasitically colonize and promote disease in other susceptible host organisms. When a microbe never or rarely causes disease, it is considered as being non-pathogenic. When it only causes diseases in situations in which the hosts have their defenses suppressed by various other stress agents, it is then called ‘opportunistic pathogen’. When it has a genetic make-up that serve to specifically facilitate the infection (virulence factors), even of healthy organisms not under any sort of stress, it is then considered a true pathogen [83]. An alternative classification though is ‘environmental pathogen’, which refers to those microorganisms that can spend part of its life cycle out of its host, but causes disease when coming into contact with it, without the need of specific conditions as in the opportunistic pathogens [11]. 

**Figure 1:**
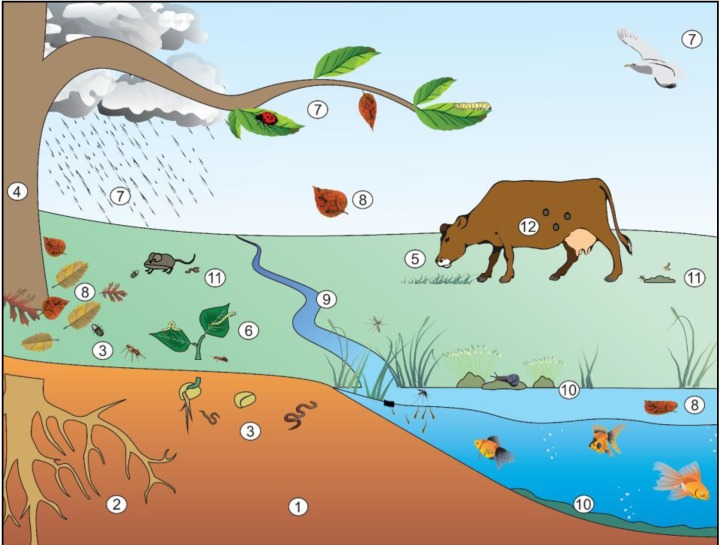
Simplified view of complex lifestyle of *Bacillus thuringiensis* (*Bt*) in different environmental niches. Vertebrates and invertebrates are only examples of a much wider range of possible hosts. The soil (**1**) is usually the largest reservoir of *Bt*, because it receives the highest amount of propagules from other environments. From it, *Bt* can colonize the rhizosphere (**2**) feeding on roots exudates. If eaten by soil invertebrates such as worms, insects and nematodes (**3**), *Bt* can infect in a paratenic way, colonizing the gut and feces, or in a pathogenic way, killing the host and growing in the cadaver. Thus *Bt* is re-introduced into the environment through these two ways. Rhizosphere colonization favors endophytic colonization (**4**) which protects the plant from some herbivores, while helps *Bt* to proliferate in plant tissues and infecting herbivores in paratenic (**5**) or pathogenic (**6**) ways. Besides endophytism, *Bt* can reach the surface of the plants from the soil due to the germination process, by splashes of rain water, and through the feces of animals that carry it, such as insects and birds (**7**). The infected fallen leaves can re-introduce *Bt* in soil and water (**8**). The rain may also carry the *Bt* to water bodies from soil and plants (**9**). In water the *Bt* can infect and proliferate in vertebrates or invertebrates and persiste in this environment by associating with substrates as aquatic plants and sediments (**10**). Faeces from animals that feed on contaminated plants or insects can serve as a source of nutrients for *Bt* growth, and they can act as a source of infection for coprophagous (**11**). It is known that ticks and mites are also *Bt* hosts (**12**), but the natural mechanism of infection is unknown. It is possible to observe a wide range of strategies for *Bt* occupy different niches and disperse in environment with or without causing disease.

For a microorganism to be considered pathogenic, it must display some specific features and abilities such as (i) occupy the same host’s niche; (ii) persist in it; (iii) overcome the host defenses; and (iv) colonize its tissues and/or impair its physiology [83]. For this, the pathogens have the so called virulence factors, which provide the mechanisms by which they can overcome the host resistance and cause disease. The life cycle of an environmental pathogen may involve different habitats, hosts and niches with different availability of nutrients, which makes it metabolically versatile [11]. This versatility is required for the movement of the pathogen through different niches and different hosts [84]. In this regard, *Bt* has several factors (such as enzymes, toxins, antimicrobial compounds and structural proteins) that allow dwelling in a diverse array of environments and reaching various target organisms/hosts (Figure 1), turning this bacterium into a well-succeeded environmental pathogen [1,6,18,34,36].

On another hand, septicemia (blood infection) usually kills the host, and the ability of a microbe to reach, grow and produce toxins in the hemolymph, causing host poisoning and death, is often associated with pathogenic organisms, not with opportunists [85]; these characteristics are widely recognized in *Bt* [2,86,87] thereby strongly pointing to it as *bona fide* insect pathogen [6]. However, another important aspect to consider in this scenario is that host specificity (due to different gut-membrane receptors, pH levels, protease activities, *etc.*), growth physiology, and secreted toxin types and amounts in *Bt* can vary substantially among strains [1,3,88], thereby interfering with a clearer understanding of its action. Moreover, *Bt* collections are frequently not properly assessed, with isolates being misclassified with regards to their toxicity when inappropriate hosts and/or toxin-secretion times for each isolate are used in the screening procedures. Methodologies have been developed to attempt screening large collections [89,90], but the lack of knowledge of possible targets, as well as suitable rearing techniques and bioassays hinder even further more appropriate assessments of *Bt* ecology concerning its pathogenicity. For instance, some studies have reported isolation of non-insecticidal *Bt* strains [26,91], but only a few species representing most relevant taxonomic orders of insects were used for the toxicity assays. Given the very wide range of possibilities for novel or different hosts for the *Bt* isolates (insects or other taxa), such a statement of non-pathogenicity might be premature and uncertain. In addition, most studies base their assessments only on the variable ‘mortality rate’ to define pathogenicity; other toxi-infection pathogenic symptoms, such as delays in development, lack of pupation, body deformations in adults *etc.*, can also be used to evaluate different types and/or intensities of pathogenic effects under play. Therefore, it appears to be very difficult to be absolutely certain about a *Bt* strain not being pathogenic in all circumstances.

#### 2.7.1. Pathogenic Arsenal of *Bt* Cells towards Insects

**Figure 2:**
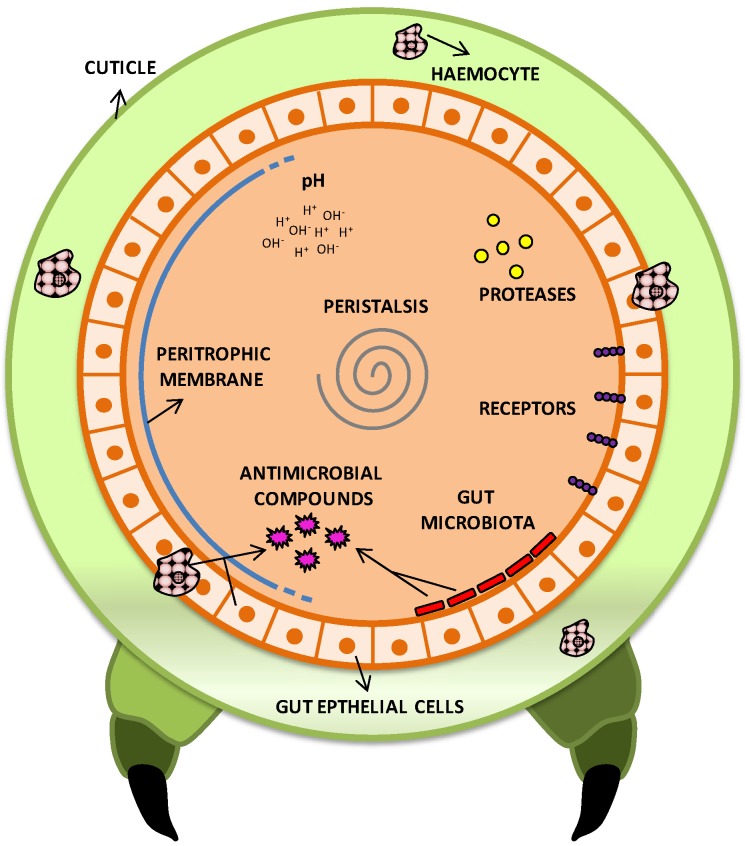
Schematic diagram of local defense mechanisms of an insect model against pathogenic bacteria*.* The cuticle is a first barrier, which can be overcome through spiracles or injury. To cause infection, ingested pathogens must overcome various physical barriers, such as peritrophic membrane, epithelium, peristalsis and commensal microbiota, as well as chemical defenses present in the digestive system as pH, proteases, cell receptors and antimicrobial compounds. In addition, the commensal microbiota provides a competitive environment for the pathogen establishment, and also produces antimicrobial compounds that hinder the pathogenic action. Much of the toxins secreted by pathogens require specific receptors to perform their functions; changes in these receptors allow development of insect resistance to pathogens. Finally, after overcoming all these defenses, the pathogen must still deal with the innate immune system and circulating haemocytes to succeed with an infection.

The insects are well studied target hosts and possess a wide range of defensive systems against pathogens. For instance, physical and chemical defenses/barriers help prevent pathogens to invade or cause damage to the insect body. The external cuticle, intestinal microbiota, intestinal peristalsis and peritrophic membrane (the inner layer of the insect midgut) are examples of physical barriers. As chemical defenses, the pH of the gastro-intestinal tract, proteases, antimicrobial peptides, cellular receptors and the immune system (Figure 2) are the main ones [6,92]. Therefore, for a microbe to cause disease, it must overcome host defenses, colonize its body (totally or partially) and elicit some physiological injury. To achieve this, it must possess molecular and biochemical resources and tools, which are usually known as ‘virulence factors’. Virulence is the measure of pathogenicity of a microorganism and reflects its ability to cause disease, even in the presence of host defense mechanisms [83]. The *Bt* species has a large variety of factors that guarantee its success as a pathogen (Table 1).

**Table 1:** Virulence strategies found in *Bacillus thuringiensis* and their roles.

Virulence strategies	Function	References
Toxins production	Degradation of intestinal membrane and release of nutrients, favoring spore germination and colonization. Gate opening to reach hemolymph and cause septicemia.	[1,2,6,66,93]
Antimicrobial resistance	Resistance to antimicrobials produced by the host and midgut resident microbiota, allowing colonization.	[94]
Antimicrobial production	Decrease of competition for space and nutrients, and reduction of antimicrobial compounds production by midgut microbiota.	[95,96]
Peristalsis and feeding reduction	Decrease of toxins and Bt cells elimination from intestine.	[6,97]
Production of degrading enzymes	Degradation of antimicrobial agents from host and facilitation of intestinal colonization. Direct toxicity in some cases.	[92,98,99,100,101,102,103]
Imune system resistance	Prevention of phagocytosis and resistance to antimicrobial compounds, digestive enzymes, pH, reactive oxygen species.	[92,104]

In a balanced symbiotic condition, microorganisms and their hosts live harmoniously, with the latter ensuring food and shelter and the former helping with protection against other pathogenic microbes [105]. However, any stress factor can initiate a pathogenic action from certain symbionts, which for example, reach alternative habitats such as migrating from intestine to the haemolymph [106]. In this scenario, *Bt* toxins can work as such stressing factors, decreasing the action of host’s natural defenses, and so, providing access of *Bt* cells and other commensal microbes from intestine to other body parts where they can cause disease. In this colonization event, the most adapted microorganisms tend to carry specific virulence factors that furnish a selective advantage towards higher proliferation rates. This establishes complex microbial interactions that can help explain more or less contribution of the resident microbiota in the mortality of organisms infected by *Bt*. These interactions, defined by host-specific composition of the intestinal microbiota, can determine the predominance of *Bt* as the main pathogen or only as a facilitator of infections by other species [95,107,108,109]. In this case, for its own proliferation, *Bt* can benefit from an eventual host death caused mainly by another microbe. Table 1 displays some strategies found for *Bt* to cope with these scenarios. 

In evolutionary terms, nonpathogenic microbes do not have to develop so many mechanisms to overcome defense systems of other organisms, since they do not belong to the same natural niche [92]. Moreover, it is of general ‘interest’ for pathogens not to kill their hosts [83], so that biotrophic parasitic activities can be maintained. In this sense, host death seems to provide the main way for *Bt* reproduction, since the host cadaver is where the greatest multiplication rate of the bacterium occurs [9,110]. However, a confounding aspect when thinking on *Bt*’s ecology is that it apparently does not have to be an obligate microbe in its association with insects, as most of pathogenic *Bt* isolates characterized thus far are capable of growing in regular media *in vitro* [12]. Moreover, *Bt* can reproduce well in other hosts (and some insects) without having to kill them (see Section 2.7.2. below). Part of the difficulty in a better understanding of *Bt*’s life style appears to be due to the fact that the vast majority of related research has focused on insect-pest species, using mortality as the single parameter of pathogenicity. More studies outside this utilitarian view are required to unravel possible alternative targets and niches occupied by *Bt*.

In the next sub-sections, the large variety of virulence factors that compose *Bt*’s pathogenic arsenal is discussed, focusing on the available knowledge about their structure, action mechanisms, molecular interactions and practical implications on biocontrol strategies and/or ecological attributes.

##### 2.7.1.1. Protein Toxins that Need Receptors in Host-Cells Membranes

The main insecticidal activity of *Bt* comes from endospore inclusions of proteinaceous crystals. These are composed of a class of proteins called *δ-endotoxins*, which are divided into two families, the Cry (crystal) and Cyt (cytolytic) proteins encoded by the respective *cry* and *cyt* genes [2,111,112]. Their ability to rapidly crystallize helps decreasing its susceptibility to premature proteolytic degradation, although there are active Cry proteins that do not crystallize, such as the Cry1I (formerly CryV) [113,114]. Cry proteins generally have three domains. Domain I is responsible for (i) the toxin insertion into the insect host’s midgut membrane; (ii) lytic pore formation; and (iii) maintenance of the toxin binding to receptors in the intestinal epithelium. Domain II is the least conserved among δ-endotoxins and is responsible for toxin binding to specific membrane receptors, playing a key role in toxin selectivity. Domain III is responsible for preserving the structural integrity of the toxin, and also for assisting in binding, penetration and pores formation in cells of the intestinal epithelium [2,115].

One of the greatest advantages of Cry toxins for development of biological control approaches is their high specificity to target host, with no significant effect to invertebrate or vertebrate non-target organisms [2]. The classical and intensively researched general mechanism for insecticidal activity as a consequence of Cry toxins action is (i) spore + crystals ingestion by a susceptible insect; (ii) crystals solubilization in the intestinal alkaline environment; (iii) action of specific proteases that cleave the protoxin into smaller active peptides—the δ-endotoxins; (iv) toxin binding to specific receptors of midgut cells; (v) membrane insertion of toxin; (vii) formation of permeable ionic pores leading to cell destruction by colloid osmotic lysis; (viii) paralysis of the insect gut and mouthparts, resulting in cessation of feeding; (ix) intestinal rupture that favors germination of the ingested *Bt* endospores; and (x) septicemia caused by the consequent bacterial proliferation. This sequence of steps and combination of factors lead to insect death in a few days [2,3]. Variations of sensitivity to intestinal pHs, types of gut proteases, and different intestinal receptors help to explain differences in the toxicity degrees among Cry proteins. Moreover, these characteristics are also considered to be the basis upon which insect resistance mechanisms can develop [2,3]. Based on all information currently available, two recently proposed theories are attempting to account for the mechanistic details of Cry toxins’ action. The first one is the *Sequential Binding Model*, which postulates that the Cry toxin must undergo proteolytic cleavages to sequentially bind to at least three different receptors; then, changes in 3-D conformation allow its oligomerization to form a pore in the cell membrane [116]. However, it is not yet fully understood whether there is a need for different receptors, and/or whether oligomeric ‘pre-pore’ structures are formed, being (or not) more active than the monomers. There is some evidence suggesting that pores are assembled only after insertion of the monomers into the membrane [112,113,114,115,117,118]. The second is the *Signaling Pathway Model*, which postulates that a signaling pathway starts in cell after Cry binding, leading to its necrotic death without necessarily forming lytic pores in the gut membranes. This model has not been easily accepted, as it does not attempt to reconcile with all research evidence strongly pointing to the formation of pores and to a direct toxicity of Cry proteins [119,120]. Further studies are yet needed to confirm, refute, or at least try to reconcile these two models, which may co-exist and complement each other (for reviews, see [6,116]).

Another type of δ-endotoxin that needs a receptor in the host gut membrane is the Cyt protein, which has a molecular weight between 25 and 28 kDa [121]. It is a cytolytic and hemolytic toxin, with primary toxic effects on dipteran insects [112,122]. These toxins have a high specificity to target hosts, being harmless to humans, other vertebrates and plants [112]. They are hydrophobic, show no homology to Cry sequences, and have different membrane receptors in the insects [2]. Their possible mechanisms of action have not been completely understood; they appear to operate in two different ways, depending on the toxin concentrations. At low levels, this toxin can develop oligomeric pores in the cell membranes of the insect gut by forming β-barrel structures [122,123]. At high concentrations, due to their high affinity to lipids, these toxins can act similarly to detergents by rupturing the cell membrane [122]. Moreover, several authors have reported a synergistic effect of Cyt with Cry toxins [19,124,125,126]. However, antagonistic effects have also been observed between Cry and Cyt proteins, especially when using toxins of different strains [127]. 

The Vegetative Insecticidal Proteins (Vip) are other promising toxins in biological control of insect-pests that require receptors in insect-gut cell membranes for their action [66,128,129]. Vips were named as such because, unlike the Cry and Cyt proteins, they are mainly produced during the vegetative growth phase of *Bt* cultures, although their secretion can also be extended into the sporulation stage [66,130]. Vip genes have shown no DNA sequences homology to δ-endotoxins, suggesting they bind to different receptors [66,130]. A practical advantage of this difference is that cross-resistance of insects to different toxins are unlikely to occur, so that Vips can be used together with Cry and Cyt proteins in biological control programs [129]. The genes encoding Vips are located in plasmids that also encode Cry proteins [131]. Four basic types of Vip toxins have been described, although they can be present in a variety of forms within each class [128,132]. Vip1 and Vip2 types have 100 and 52 kDa, respectively [133], and form a pair of binary toxins, where Vip1 binds to specific receptors on the intestinal membrane, creating pores through which Vip2 penetrates and causes inhibition of the polymerization of monomeric actin (G-actin) [133,134]. Its main effect is against insects of the Coleoptera order [133]. Vip3 toxin has 88.5 kDa and is effective primarily against lepidopteran insects [66,133], with a mechanism of action apparently similar to that of Cry toxins, by forming pores in midgut cell membranes of the target-pest that cause osmotic imbalance and degeneration of the epithelial layer [128,129]. Vip3 also appears to do not show deleterious effects to non-target organisms [135]. Vip4 was not characterized until now. 

##### 2.7.1.2. Proteins and Toxins that Do Not Need Receptors in Host-Cell Membranes

The α-exotoxins (a.k.a. phospholipase-C) have been purified from culture supernatant of certain *Bt* strains [98]. They are thermolabile proteins classified according to the types of phospholipids onto which they act; for example, phosphatidylcholine-specific phospholipase-C (PC-PLC) [136], phosphatidyinositol-specific phospholipase C (PI-PLC) [137], *etc.* Their toxicity can be explained by the hydrolysis of various phospholipids on cell membranes [138], being highly toxic to insects by oral or intra-haemocoelic administration. They cause degeneration and lysis of insect cells such as the haemocytes [139] and, at high doses, can also cause toxicity against vertebrates [140].

Chitin is a long-chain polymer of *N*-acetylglucosamine (a glucose derivative), and is the main component in the cell walls of fungi, exoskeletons of arthropods, and midgut peritrophic membrane of many insects. Endochitinases are enzymes that degrade chitin by randomly cleaving within its chain, and exochitinases hydrolyze diacetylchitobiose units from the chain’s end [99,141]. Pathogens that produce chitinases have a great advantage over host defenses, with some *Bt* strains showing to produce both forms of chitinase simultaneously [99]. It has been proposed that *Bt* can degrade the perithrophic membrane to facilitate binding of other toxins to their receptors in midgut epthelium [142]. To support this view, some researchers have shown that the use of both exogenous [99,143] and endogenous [99] chitinases has increased the efficiency of Vip and Cry toxins. It appears, therefore, that the combined use of usual *Bt* toxins and chitinases can increase success in pests biocontrol programs.

Further types of toxins have also been described for *Bt*. For instance, hemolysins are also produced during *Bt* vegetative growth phase, especially when iron is depleted. Its mechanism of action is based on the lysis of insect haemocytes and macrophages by forming pores in the cell membranes [102]. Essentially, they allow the *Bt* cells to obtain nutrients during the infection, while repressing the host immune system. 

Li and Yousten [144] first described the production of a *Bt* protease, specifically the metal chelator-sensitive protease (metalloprotease), which was shown to be secreted near the stationary phase of growth. The required proteolytic cleavage of Cry toxins for their activation [2,3] depends on not only the action of exogenous proteases, present in the host midgut, but also on the action of various *Bt*-synthesized endogenous proteases [103,144,145]. A metalloprotease named InhA was shown to be involved in the toxicity of *Bt* against Lepidoptera [100], while the Bmp1 showed activity against Nematoda [103]. In addition to participating in the activation of Cry toxins, the metalloproteases can also protect *Bt* from the innate insect immune system through cleavage of antimicrobial peptides, or by facilitation of the bacterial cells’ escape from the haemocytes [104,146]. The destruction of cells and tissues to facilitate *Bt* colonization of the host body is another form of proteases action [92,103].

Finally, secreted insecticidal proteins (Sip) were discovered and characterized by Donovan *et al.* [147]. This protein is secreted during the vegetative phase of *Bt* growth and was identified as toxic to coleopteran larvae. Its basic mechanism of action is similar to Cry and Vip toxins, *i.e.*, by forming pores in cell membranes that disrupt the regular physiology of the insect.

##### 2.7.1.3. Non-Proteinaceous Toxins

The type I β-exotoxin (a.k.a. ‘thuringiensin’) is a thermostable toxin of a low molecular weight (701 Da), composed of adenosine, glucose and alaric acid. It is an adenine nucleotide (ATP) analog [148] that acts by inhibiting eukaryotic DNA-dependent RNA polymerases, thereby affecting the normal development of the organism [149]. Due to this general mechanism of action, a very high toxicity was observed against a diverse array of *taxa*, including mammals and other vertebrates, so that the World Health Organization has recommended that *Bt* strains producing these toxins must not be used for insect control [150]. Some countries, however, still use products based on β-exotoxins, mainly in specific control programs of dipteran pests that are resistant to other insecticides [151]. Ohba *et al.* [152], studying other *Bacillus* species (*B. subtilis*, *B. megaterium* and *B. natto*) have not identified the β-exotoxin production, suggesting this feature is strain-specific to *B. thuringiensis* and *B. cereus*. It is known that the phosphate group present in the β-exotoxin’s structure is essential to its toxicity. Thus, the cleavage of the phosphate ester when treating with phosphatase [149], or heating at a pH < 3 [153,154], causes phosphate removal, and so, the loss of the toxic activity. Genes that control the synthesis of β-exotoxin seem to be located in plasmids that also encode Cry proteins [153,155]. This may explain why it is secreted near and during the sporulation phase, along with the Cry toxins. Another form of β-exotoxin molecule identified (type II) is analogous to the uracil nucleotide (UTP) and seems to be the most toxic, being particularly effective against insects of the order Coleoptera [153]. Its whole structure, however, is not yet fully known. 

Taking together all virulence features described above, it becomes readily apparent the extraordinary genetic plasticity found among specimens of this bacterial species, being clear-cut evidence towards a characteristic fast pace for its evolutionary path. However, the existence of a toxin with lack of host-specificity, but not present in all studied strains, poses a further puzzle to be solved in the understanding of *Bt*’s ecology related to pathogenicity: why evolving such a sophisticated mechanism of overcoming defenses to kill the host (through Cry, Cyt, Vip and Sip toxins, as well as other proteinaceous enzymes that aid in the infection), if non-specific action in killing any type of host would be apparently more advantageous for occupation of various niches by *Bt*? Perhaps it may represent ancient steps in the co-evolution of insects’ specificities; its presence in some strains may represent ‘live fossils’ of primitive mechanisms of host killing. Alternative, it may have simply been acquired through horizontal transfer and being maintained in some strains as a way of helping the bacterium with some levels of proliferation and dispersion through indistinct transfer among a variety of hosts. Further studies based on these hypothesis need to be devised to answer this question.

#### 2.7.2. Pathogenicity to Other Taxa

The fact that *Bt* has been found in places apparently free from insects [12] is likely related to its dispersion capacity in the environment (wind, rain, paratenic hosts feces, *etc.*—Figure 1), or yet, to the presence of other host *taxa* that were not yet identified and/or assessed. Besides insects, other *taxa* have been described as being targets for *Bt*. Thus far, high levels of *Bt* toxicity have also been found against ticks, mites, nematodes and protozoans, mostly from studies aiming at searching for appropriate biocontrol agents. 

The tick (Arachnida: Acari) is a major ectoparasite that affects animals, causing several zoonoses of economic relevance. Its control is achieved by synthetic acaricides. Although research on the biological control of ticks has been mainly focused on fungal agents, some work has been done with bacteria; *B. thuringiensis* subspecies *kurstaki*, *israelensis* and *thuringiensis* have shown mortality and development retardation of soft and hard ticks, including their eggs [156]. *Bt* kurstaki’s toxins were effective against hard ticks by spraying [157,158] and immersion [159]. It has also been found that toxic effects are the greatest during critical physiological stages such as egg, pupation and metamorphosis. These data also suggest that there might be another form of infection beyond the intake, perhaps through other natural openings in the body (e.g., genitals and spiracles) [6], thereby pointing to the possibility of alternative mechanisms of pathogenicity. *Bt* has been isolated from the hemolymph of dead ticks, proving the existence of a systemic infection [156]. It was demonstrated that some *Bt* toxins are lethal to hemoplast cells and that Cry4Ba was capable of binding receptors, forming pores and lysing the cells [160]. Besides ticks, pathogenicity of *Bt* has also been reported against storage mites (Arachnida: Acari), particularly by intake [161,162]. 

The Nematoda species are cylindrical and elongated multicellular animals that can be either free-living or animal and plant parasites. Because of its economic importance, its biological control has been investigated. Some studies involving *Bt* toxins have demonstrated their effectiveness against this parasite, including both Cry [163,164] and vegetative proteins from the culture supernatant [164]. The Cry5, Cry6, Cry12, Cry13, Cry14 and Cry21 proteins were described as nematicidal or developmental retardant [3,163]. The mechanism of Cry toxins action in nematodes is similar to that on insects, correlating with damage to the intestine. Symptoms of nematode poisoning include lethargy, reduced size, pale coloration and contraction, vacuolation and degeneration of the intestine [163]. 

Protozoa are unicellular eukaryotic microorganisms present preferably in moist environments. They may be free-living or animal parasites, including humans. The first report of a *Bt* strain able to control infections by a protozoan was made by Thompson and Gaertner [165]; subsequently, other strains were identified as effective in the control of *Trichomonas vaginalis* [166]. It has also been reported that *Bt*, after ingested by *Tetrahymena pyriformis* and encapsulated in food vacuoles, was able to multiply and sporulate, producing δ-endotoxins that remained protected, stable and active. This behavior demonstrates that protozoans can act at least as an intermediate host, being possibly part of the natural recycling and transmission of *Bt* [80].

In our view, two main lessons can be learned from these research: the first is that conclusions about the lack of hosts for *Bt* isolates in given areas must be always considered with care, as potential true hosts may simply be there but not aware of; the second is that mortality should not be the only (or main) parameter to use for assessment of pathogenic effects of *Bt*, as different forms or levels of toxicity that affects development and/or reproduction of hosts can be both ecologically informative and agronomically useful.

## 3. Reconciling Alternative Ecological Views

To achieve this task, it seems necessary to take some distance from the utilitarian view of *Bt* as a bioinsecticide/biocontrol agent, as this tends to make us overlook the behavioral and functional complexity of this bacterial species. A multidisciplinary and integrated view involving concepts from microbiology, epidemiology, infectology, toxicology, development, ecology, genetics, genomics and evolution is required to provide a more suitable framework for the debate on *Bt*’s role in nature. This approach inevitably leads us to recognize that perhaps the choice of a single line of thoughts—“saprophytic *Bt*”, “pathogenic *Bt*”, “free-living *Bt*”, *etc.*—is likely insufficient to account for all related aspects already found and reported in the literature. We argue that all the ideas and concepts currently available about *Bt*’s ecology actually complement each other and points to an extreme versatility of this bacterial species in occupy an array of different niches. The *bona fide* pathogenicity of *Bt* on various insect taxa has already been more than sufficiently proved, as it is a fact that host-specificity is achieved by different combinations of elements from a multifaceted arsenal of toxins and strategies to overcome host defenses. In evolutionary terms, this integrated view of a functionally versatile microbe implies that *Bt* should have a fast rate of changes in its genome, both through accumulated mutations (reasonable to be expected for a short living-cycle organism) and incorporation of new genes (through conjugation, transformation and transduction). Fang *et al.* [15] have recently demonstrated that *Bt* shows an ‘open pangenome’ as a key evolutionary characteristic, which favors its adaptation through the mechanisms just mentioned, as opposed to another closely related *Bacillus* species, the *B. anthracis*, which appears to have a ‘closed pangenome’ that tends to restrict its ecological niches. Conjugation of *cry* genes are thought to be responsible for the high degree of diversity in Cry proteins; such an ever increasing diversity also suggests a rapid evolutionary pace for this species, and ensures high levels of adaptation for *Bt* strains to their target organisms [167].

Considering the vast amount of work done on the aspects related to *Bt*’s capability as a biological control agent (*i.e.*, about its pathogenic behavior against mostly insect-pests), it is important to realize that such abilities may be intimately associated with human interference on Earth’s face. Human-related changes in the environment began more noticeably with the onset of civilization and agriculture at the Neolithic era, ~12,000 years ago [168], with practices related to clearing vegetation and opening areas for crop cultivation and cities construction, as well as related to destructions caused by war movements and battles. Since then, and as largely acknowledged today, crop cultivation in cleared areas narrows down the genetic background and trophic interactions, so that selective pressure favors few species that circumstantially become most adapted, such as insects, nematodes and others. Therefore, it appears conceivable that evolution of *Bt* as an insect pathogen (occupying agricultural and other man-affected niches) has long been favored by a direct human interference in the environment. Nevertheless, as an environmental bacterium occupying a variety of niches, with very many ways to disperse, *Bt* is capable of grow vegetatively (although slower and for few generations if not on other animals) and survive adverse conditions for long periods through sporulation. In fact, it can be logically posed that *Bt* populations dwelling in the environment had probably been the major evolutionary raw material (ancestors) for development of specifically adapted insect-pathogen strains found later on. Likely, this process is yet ongoing around the globe, generating a vast array of different strains, and so, justifying the ever continuing research for novel strains and toxins with biocontrol capabilities [28,62,88,89,90,169]. In this scenario, it is relevant to note that studies have shown no differences in competitive ability in plant colonization between strains with and without production of Cry proteins [34], suggesting that the metabolic burden of insecticidal crystal proteins (ICP) production apparently does not significantly reduce fitness. Taking all these ideas into account, the ecological role of *Bt* appears to be as multi-host-environmental-pathogen [11,170].

Several factors may be involved in the ability of infecting multiple hosts. As discussed above, mutations can alter existing genes and grant them new and/or improved functions [171], as well as the conjugation of *cry* genes that widens the range of possible hosts [167]. Another factor is host speciation, in which a microorganism affecting ancestor species can also infect their derived species [172]. Genetic similarity among different host species also influences the success of a multiple-infection pattern, since the related defenses, metabolism, niches and habits are similar, thereby favoring a faster adaptation by the pathogen [170]. Finally, the introduction of exotic hosts in areas inhabited by the pathogen, and vice versa, can promote successful infection if the host does not offer resistance by not having come into previous contact with the pathogen [173]. Being capable of infecting various hosts and inhabiting different environments multiplies the chances of *Bt* in succeeding at survival and proliferation.

## 4. Conclusions and Perspectives

After over 100 years of *Bt* discovery, new target species have constantly been found and its mechanism of action is yet under discussion. With the studies conducted to date, we realize that different strains of *Bt* appear to have co-evolved with different taxa, making it difficult to clarify the real ecology of such a highly versatile bacterium. Although it is currently well acknowledged by the scientific community that *Bt* is a pathogenic bacteria to insects, little has been studied and discussed about the interactive patterns of *Bt* with other taxa and the ecological relevance of them. Therefore, a discussion still remains on whether *Bt* has a role in nature as an opportunistic pathogen, and/or a saprophyte, and/or simply as a cosmopolitan microbe that occupies an array of different niches with various efficient forms of dispersal. The not so many studies available that directly addressed this issue, as well as inherent difficulties recognized in this type of research, have hindered a better clarification over what is the real ecology of this bacterium. Part of this scenario is also explained by the fact that the majority of studies to date have focused on (and so, limited to) pest-insects of agricultural or public health relevance, thereby leaving important gaps on the knowledge about *Bt* interactions with other organisms/hosts. 

In this review, we have intended to collect and integrate available and relevant information in this context, perhaps a bit overlooked thus far. Based on the available studies, we have shown that *Bt* is capable of being present, both in vegetative and sporulated forms, in a variety of environments without the need of hosts; however, in each environment, it can reach hosts from a diverse set of *taxa*, and colonize their bodies in the pathogenic or paratenic manner, as well as their cadavers and excrements. Hence, *Bt* displays a high capacity of proliferation and dispersion, mostly due to its combined ability of grow and produce spores almost everywhere, and passively reach all sorts of environments. Coupled with a reasonable genomic plasticity, rapid co-evolutionary events have changed some of these isolates into host interactive ones that became pathogenic at various levels. The overall *Bt* classification remains open until more conclusive studies are reported, which should consider such a great genetic plasticity, spectrum of action, possibility of paratenic and intermediate hosts, and versatility to occupy different niches, all within more integrative approaches*.* The current available knowledge strongly leads us to accept a broader idea that *Bt* is a multi-host-enviromental-pathogen, or simply an enviromental-pathogen. Further ecologically-oriented research on *Bt* will benefit from basing the generation of working hypotheses on this point of view, rather than on the predominant view of it as a potential biocontrol agent. 

It is also worth mentioning, as a next step for research, that some *Bt* strains produce crystalline parasporal proteins during sporulation that, until today, have shown to be non-toxic to insects and to differ from other Cry and Cyt proteins. Mizuki *et al.* [174] first described the cytotoxic action of these proteins on human cancer cells. A year later these proteins were collectively named as ‘parasporins’ [175]. Although invertebrate targets for them have not been identified so far, these proteins share the same nomenclature of Cry proteins. To date, there are six parasporin families described (PS1–PS6), containing 19 different proteins according to their genetic homology. Recent work have shown cytotoxic activity of parasporins against nine human cancer cell lines and, with less intensity, against five types of normal human cells [176,177,178,179,180]. Katayama *et al.* [177] have claimed that the PS1 is not a pore-forming toxin, at least in mammal cells. It appears to act by inducing apoptosis through decreasing the levels of DNA synthesis and cellular proteins, and by increasing the levels of intracellular Ca^2+^. The formation of pores in the plasmatic membrane of the cells under the action of PS2 or PS3 has been observed [178,179,180,181]. No information is available about the mechanism of action of other parasporins. Taking these data together, strains carrying parasporins may represent novel species within the *B. cereus sensu lato* group, requiring more in-depth taxonomic studies to sort this out. The true ecological function of parasporins is yet unknown. However, one might speculate that, due to their toxicity against cancerous white blood cells, parasporins can attack host-defense cells in a similar way to what occurs with hemolysins; or yet, its function might be related to β pore-forming toxins (β-PFT), due to its homology to those toxins from *Aeromonas hydrophila* and *Biomphalaria glabrata* [182,183].
